# Phenotypic characteristics of the mycelium of *Pleurotus geesteranus* using image recognition technology

**DOI:** 10.3389/fbioe.2024.1338276

**Published:** 2024-06-17

**Authors:** Xingyi Wang, Ya Xu, Xuan Wei

**Affiliations:** ^1^ College of Mechanical and Electronic Engineering, Fujian Agriculture and Forestry University, Fuzhou, China; ^2^ College of Computer and Information Sciences, Fujian Agriculture and Forestry University, Fuzhou, China

**Keywords:** image recognition, mycelial quality, outline, phenotypic characteristics, *Pleurotus geesteranus*, texture feature

## Abstract

Phenotypic analysis has significant potential for aiding breeding efforts. However, there is a notable lack of studies utilizing phenotypic analysis in the field of edible fungi. *Pleurotus geesteranus* is a lucrative edible fungus with significant market demand and substantial industrial output, and early-stage phenotypic analysis of *Pleurotus geesteranus* is imperative during its breeding process. This study utilizes image recognition technology to investigate the phenotypic features of the mycelium of *P. geesteranus*. We aim to establish the relations between these phenotypic characteristics and mycelial quality. Four groups of mycelia, namely, the non-degraded and degraded mycelium and the 5th and 14th subcultures, are used as image sources. Two categories of phenotypic metrics, outline and texture, are quantitatively calculated and analyzed. In the outline features of the mycelium, five indexes, namely, mycelial perimeter, radius, area, growth rate, and change speed, are proposed to demonstrate mycelial growth. In the texture features of the mycelium, five indexes, namely, mycelial coverage, roundness, groove depth, density, and density change, are studied to analyze the phenotypic characteristics of the mycelium. Moreover, we also compared the cellulase and laccase activities of the mycelium and found that cellulase level was consistent with the phenotypic indices of the mycelium, which further verified the accuracy of digital image processing technology in analyzing the phenotypic characteristics of the mycelium. The results indicate that there are significant differences in these 10 phenotypic characteristic indices ( P<0.001 ), elucidating a close relationship between phenotypic characteristics and mycelial quality. This conclusion facilitates rapid and accurate strain selection in the early breeding stage of *P. geesteranus*.

## 1 Introduction

*Pleurotus geesteranus*, a globally cultivated edible fungus, plays a significant role in economies worldwide (Singh et al., 2021; Wei et al., 2023). Analysis of *P. geesteranus* market prospects in 2024 showed that the global *P. geesteranus* market size is approximately 10.9 billion US dollars and is expected to grow at an annual rate of about 5%. In 2023, *P. geesteranus* accounted for 17.45% of the output of edible fungi in China. The seeds of edible fungi are referred to as edible fungus strains. According to the source, generation of propagation, and production purposes, they can be divided into three levels: mother strain, original strain, and cultivation strain. The mycelial quality is intricately tied to its yield, making it a crucial factor influencing economic benefits (Zhang et al., 2019). Mycelial degeneration refers to the phenomenon where the traits of edible fungus populations deteriorate, leading to deviations in yield, quality, and resistance that do not meet human needs. Mycelial degeneration is a serious issue commonly encountered in the cultivation of various edible fungi. In practical production, mycelial degradation generally takes place during its preservation and subculture. Using degenerated strains for cultivation often results in delayed fruiting, low yield, and poor quality, which cause significant economic losses to producers of edible fungus (Pérez et al., 2021). The mycelial phenotype is the collective expression of its individual traits within a certain environment and provides fundamental information for breeding purposes (Helgason and Fitter, 2009). Mycelial degeneration of *P. geesteranus* is both complex and varied. To optimize the high production cultivation of the mycelium of *P. geesteranus*, breeders need to evaluate the mycelial phenotype by gathering phenotypic information through sampling, measurement, observation, and calculation techniques (Lehmann et al., 2019; James et al., 2022). Phenotypic parameters including mycelium growth rate, density, color, and content of specific substances such as polysaccharides, active proteins, and other bioactive components of the strain serve as the primary criteria for assessing the mycelial quality of *P. geesteranus* (Yang et al., 2022). In the initial stages of cultivation, accurately distinguishing the degenerated mycelium based on phenotypic characteristics is crucial (Danner et al., 2023). This process aids in effective selection and cultivation of the mycelium, while also preventing the degenerated mycelium from flowing into the production process, ultimately enhancing overall economic efficiency.

To date, the phenotypic characteristics of edible fungi have been assessed through observation, resulting in drawbacks like low precision, inadequate standardization, time-intensive procedures, and high-intensity labor (Furbank and Tester, 2011; Yin et al., 2022; Liu et al., 2023). Digital image processing is capable of precise computation for qualitative and quantitative analyses, instead of manual measurement, which often relies on subjective judgment based on experience. Recently, digital image processing has emerged as a vital analytical method for evaluating the phenotypic attributes of crops, encompassing factors like crop color, growth, density, and uniformity (Mahajan et al., 2015; Zhao et al., 2019; Omari et al., 2020). This approach involves analyzing pixel attributes and inter-pixel attributes and analyzing the dynamic characteristics of their trait expression over growth time (Chen et al., 2014). Guo et al. (2018) extracted a total of 51 image-based traits from 507 rice samples for studying the plant drought tolerance traits and assisting in drought-resistant breeding. Li B. et al. (2020) conducted a systematic analysis of 119 image-based digital traits of 200 cotton seedlings under drought stress. Li H. et al. (2020) quantified the correlation among 43 phenotypic characteristics of rapeseed with their growth conditions and predicted the final rapeseed yield. Currently, researchers have attained high-throughput, high-precise, and non-destructive acquisition of crop phenotypes for staple food crops like rice, wheat, and soybean, utilizing digital image processing technologies. This motivates the focus toward digital and efficient breeding research (Shakoor et al., 2017).

The phenotypes of edible fungi associated with mycelial quality are varied and intricate, encompassing factors such as growth rate, density, color, and content of specific substances. These phenotypic characteristics have a strong correlation with mycelial quality (Yoo et al., 2019). Specifically, it is generally manifested in the following aspects: 1) mycelial morphology: the relation between mycelial morphology and quality lies in the fact that the more regular and faster-growing species often have higher quality; 2) mycelial density: the higher the mycelial density, the more vigorous the mycelial growth and the higher the yield; 3) mycelial growth rate: the mycelium with faster growth rates can quickly occupy growth space and reduce competitive pressure, thereby improving yield; 4) mycelial color: the color of the mycelium can reflect the metabolic state and growth environment. The mycelium with bright and uniform coloration usually grows vigorously.

At present, the current research and applications of digital image processing for phenotypic analysis of *P. geesteranus* remain open. The objective of this paper is to investigate the inherent relations between the phenotypic characteristics of *P. geesteranus* and its mycelial quality. We acquired imaging data depicting four groups of the mycelium of *P. geesteranus*, encompassing both non-degraded and degraded states. We extracted two pivotal phenotypic characteristics of the mycelium, outline and texture, including a total of 10 indices and carried out quantitative calculation and analysis through digital image processing technology (Williams et al., 2013; Minervini et al., 2014; Yang et al., 2019; Basak et al., 2021).

Additionally, in order to study the correlation between phenotypic traits and mycelial quality, we also compared cellulase and laccase activities for verification. Cellulase, known as carboxymethyl cellulase (CL), is present ubiquitously in bacteria, fungi, and animals (Ghose, 1987). CL facilitates the degradation of carboxymethyl cellulose and finds extensive applications in medicine, food, cotton spinning, environmental protection, and renewable resource utilization (Khan et al., 2023). When cellulase breaks down cellulose, it is converted into glucose units and other reducing sugars. These reducing sugars can react with an anthrone reagent to form a blue–green complex, the intensity of which is proportional to the concentration of the reducing sugar. By measuring the color intensity after the reaction, the activity of cellulase can be indirectly calculated, that is, the amount of reducing sugar produced by cellulose breakdown per unit time. Therefore, we used the anthrone colorimetric method to analyze the cellulase activity on the mycelium. Laccase is a copper-containing polyphenol oxidase, belonging to the cupric blue oxidase family, widely distributed in fungi and higher plants, with strong redox ability, and has a wide range of applications in pulp biobleaching, degradation of environmental pollutants, lignocellulosic degradation, and biological detection.

Finally, the paper concluded that the phenotypic indices of cellulase and the mycelium were consistent and analyzed the internal relationship between mycelial quality and these phenotypic characteristics concerning the mycelial outline and texture.

## 2 Materials and methods

### 2.1 Strain image materials

#### 2.1.1 Mycelium of *P. geesteranus*

This study selected the mycelium from *P. geesteranus* (Xiu 2), along with subcultures up to the 5th and 14th generations, as well as degraded strains. All these materials were sourced from the Fungi Research Center of Fujian Agriculture and Forestry University. Among them, the non-degenerated strain was labeled as X2-N, the 5th generation subculture strain was labeled as X2-5, the 14th generation subculture strain was labeled as X2-14, and the degenerated strain was labeled as X2-T.

**FIGURE 1:**
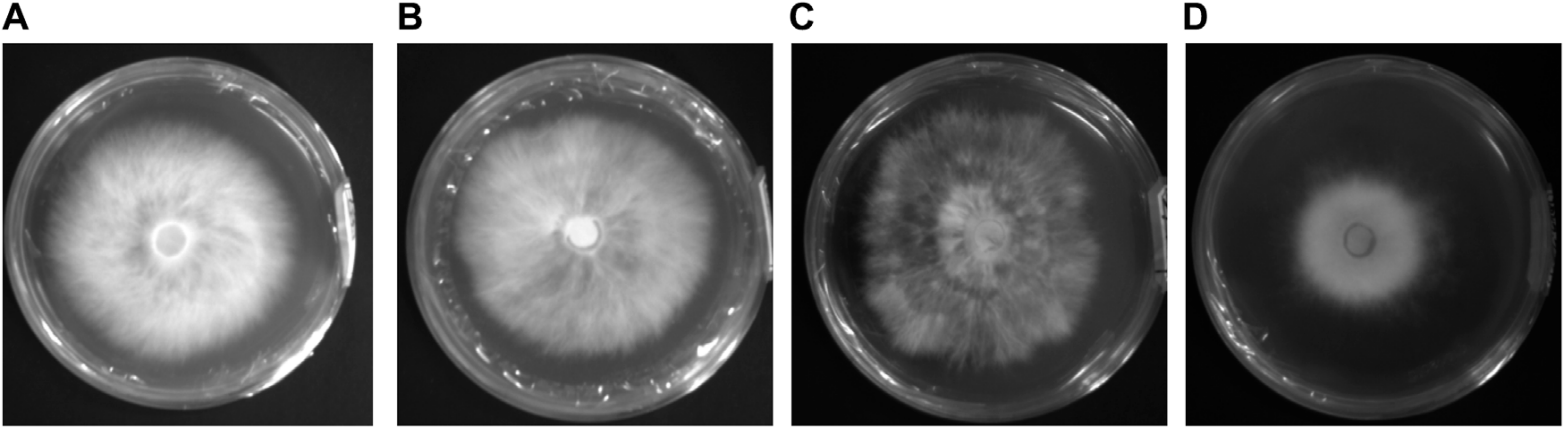
Image of four activated mycelium samples of *Pleurotus geesteranus*. **(A)** non-degenerated strain labeled X2-N; **(B)** 5th generation subculture strain labeled X2-5; **(C)** 14th generation subculture strain labeled X2-14; **(D)** degenerated strain labeled X2-T.

During the experiment, the solid medium, Potato Dextrose Agar (PDA), comprised 200 g potato, 20 g glucose, 20 g agar, and 1,000 mL water. First, we performed strain activation by inoculating each of the four mycelium strains into 90- mm -diameter Petri dishes containing the PDA medium. These dishes were then placed in a 25 °C incubation room and cultured in an inverted position for 8 days. The images of the four activated species are shown in Figure 1. In the next step, we expanded the culture on the activated strain samples of *P. geesteranus*. A 5-mm-diameter piece of the mycelium was cut from the end of the mycelium and inoculated at the center of a new 90- mm -diameter PDA Petri dish. They were cultured in an inverted position in a 25- °C incubation room for 8 days, and data were collected at that time. In this test, 100 non-degenerated strains, 100 X2-5 strains, 100 X2-14 strains, and 100 degenerated strains were collected; a total of 400 samples were preserved in the Key Laboratory of Agricultural Information Perception Technology, Qishan Campus, Fujian Agriculture and Forestry University.

#### 2.1.2 Image acquisition

**FIGURE 2:**
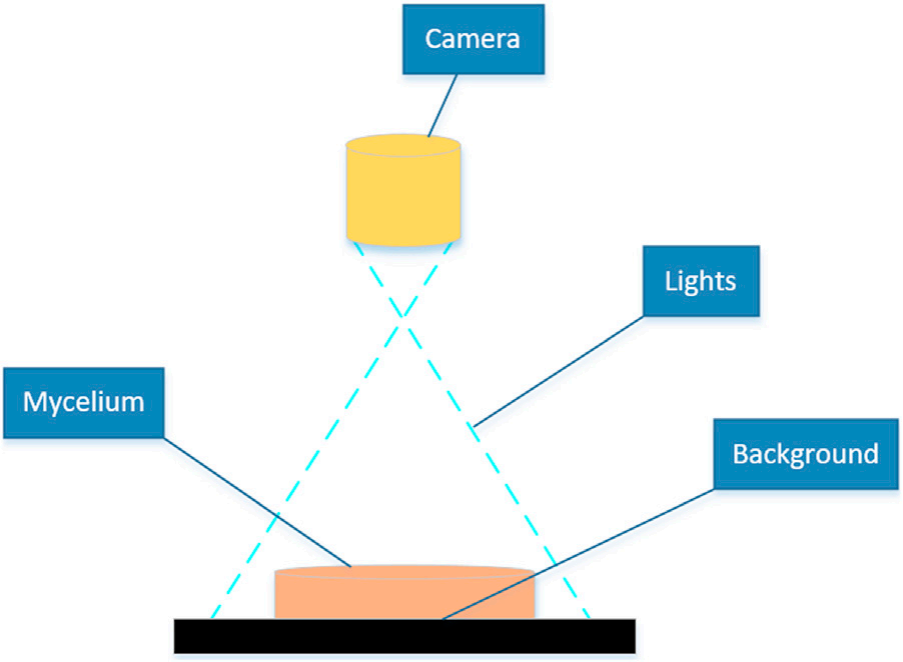
Equipment for mycelium image acquisition.

This experiment captured the images of the mushroom by using an industrial camera (Basler acA 1300-30 gm; Germany), which was equipped with a Sony ICX445 CCD chip, 20 frames per second, resolution of 1.3 million pixels through Pylon Viewer 64-Bit software. The camera was positioned at a fixed distance of 25 cm directly above the Petri dish, resulting in mycelial images with a size of 1,280 × 980 pixels. Images were captured on days 2 and 8 following mycelial subculture. The mycelial samples displaying evident abnormalities in the outline and texture morphology were excluded, and the remaining 400 samples were employed for calculation and analysis in this study. The device for image acquisition of the mycelium was set in the Key Laboratory of Agricultural Information Perception Technology at Fujian Agriculture and Forestry University, as shown in Figure 2, which consisted of an industrial camera, light source, computer, and detection objects.

#### 2.1.3 Image processing software

In order to realize the analysis and quantitative calculation of mycelial images on outline and texture indices, this experiment first used MATLAB 2020b (9.9.0.1467703) 64-bit (win64) software to perform the outline and texture processing, allowing computation in the formulations of 10 indices, namely, radius, perimeter, area, growth rate, change speed, mycelium coverage, integrity, groove depth, density, and density change. Furthermore, IBM SPSS Statistics R26.0.0.0 64-bit was utilized to analyze and optimize the normality and correlation of the acquired image data. In addition, OriginPro 2022 software outputted the statistical maps of the data. The computer environment was Windows 10 with the graphics card driver being version 27.21.14.5167 of NVIDIA GeForce GT 730.

### 2.2 Strain image processing methods

**FIGURE 3:**
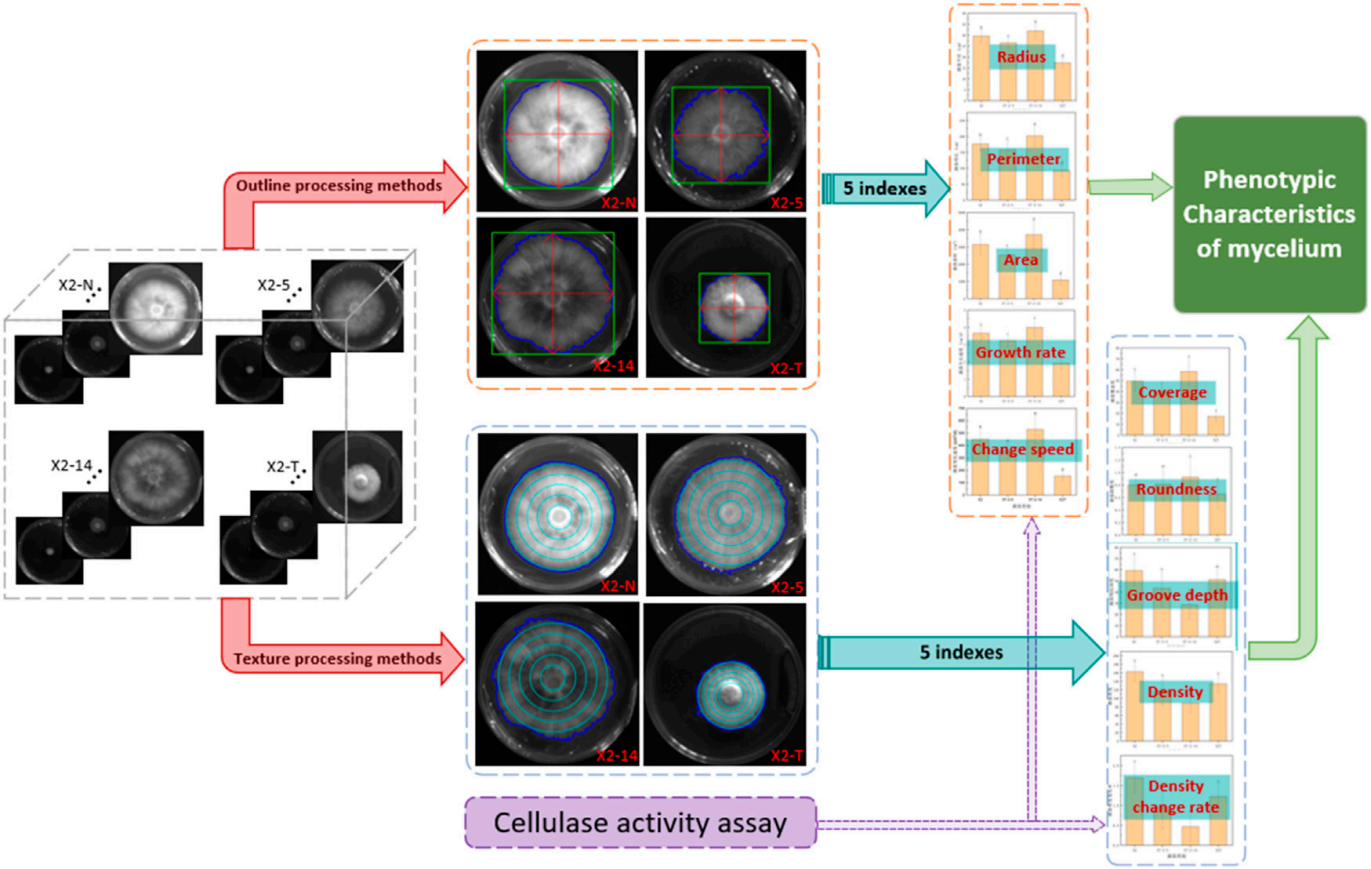
Comprehensive technical framework for phenotypic characteristics of the mycelium. 1) Four categories of mycelium samples, non-degraded (X2-N) and degraded (X2-T) mycelium and the 5th (X2-5) and 14th (X2-14) subcultures, were processed using two methods: contour and texture; 2) the calculations generated 10 corresponding indices of mycelial phenotypic characteristics; 3) statistical analysis of these data revealed the relationship between mycelial phenotypic characteristics and its quality; 4) the cellulase activity assessments were used to verify the accuracy of the above two methods.

The comprehensive technology is presented in Figure 3. We obtained 10 phenotypic indicator datasets for each strain sample by employing both outline and texture processing methods. Initially, in the outline formulation, binarization and threshold segmentation were employed to delineate the mycelial boundary, minimum circumscribed rectangle, and horizontal and vertical axes (Chen et al., 2008; Underwood et al., 2017). Following this, five indices, namely, mycelial perimeter, radius, area, growth rate, and change speed, were calculated to scrutinize the morphological and phenotypic traits of the mycelial outline (Bae et al., 2011; Le et al., 2012). Subsequently, the gray-level co-occurrence matrix was used to extract pixel count, contrast, and entropy (Xuan et al., 2022; Wu et al., 2023). An additional set of five indices, namely, mycelium coverage, roundness, groove depth, density, and density change, were calculated to analyze the phenotypic characteristics of the mycelium texture (Hu et al., 2022). Moreover, the cellulase and laccase activity indexes of the mycelium were compared, providing additional validation for the analysis results on the phenotypic characteristics of the mycelium (Samy et al., 2022). Finally, the study analyzed the internal correlation between the outline and texture concerning the phenotypic characteristics of the mycelium for *P. geesteranus*.

#### 2.2.1 Outline processing methods

**FIGURE 4:**
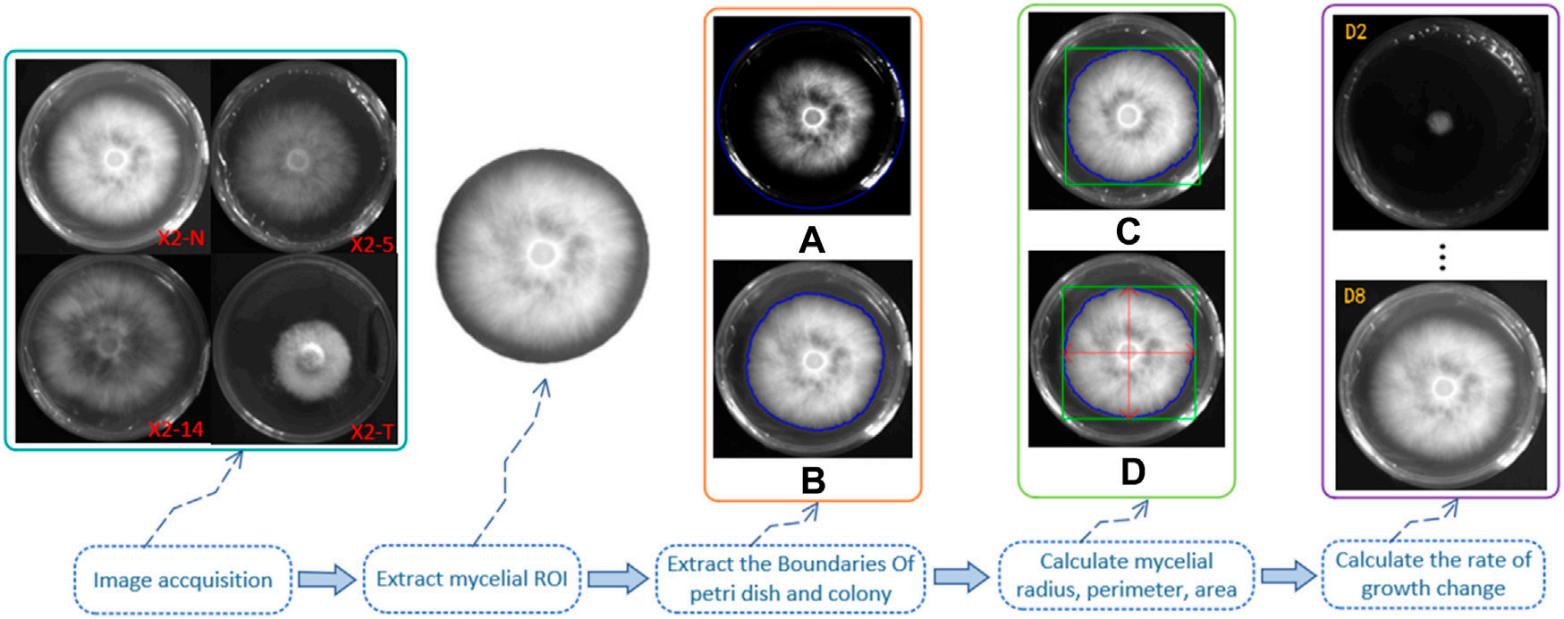
Calculation steps and methods of the mycelial outline. 1) Grayscale of the digital image of the mycelium was obtained using the camera, and the region of interest (ROI) was extracted; 2) outline of the boundary of the Petri dish **(A)** and the mycelium **(B)**, as shown by the blue line; 3) the minimum external matrix of the mycelial boundary is denoted by the green line **(C)**; 4) the radius, perimeter, and area were calculated through the horizontal and vertical axes by the red double-headed arrow **(D)**; 5) days 2 and 8 of mycelium growth were set as two unit time observation points to calculate their growth rate and change speed.

We made observations at two time points—on day 2, following mycelium activation, and on day 8 of its growth—calculating the growth rate and change speed accordingly. The calculation steps and methods are shown in Figure 4.

The detailed procedure for analyzing the phenotypic characteristics of the mycelium concerning outline morphology is summarized as follows:

Algorithm 1. The outline processing methods.

**Input:** The gray value of the mycelial image
1) Grayscale processing on the mycelial image:
 
$n_{p}$ ← Analyze the gray-level difference of the inner and outer edges of the Petri dish, and calculate the number of pixels on the border of the Petri dish;
 
$n_{v}$ ← Analyze the difference between the gray level of the mycelium and the background pixel, and calculate the number of pixels on the border of the mycelium.
2) Use the number of all 2D pixels contained within the boundary:
 
$N_{P}$ ← Calculate the number of Petri dish pixels;
 
$N_{V}$ ← Calculate the number of mycelium pixels.
3) Calculate the minimum circumscribed circle matrix:
 
$Z_{L}$ ← Extract the horizontal axis in the mycelium outline, and calculate the number of pixels on the horizontal axis of the mycelium;
 
$Z_{S}$ ← Extract the mycelial vertical axis, and calculate the number of pixels on the vertical axis of the mycelium.
4) Day 2 of strain growth was regarded as the first observation time point $t_{1}$ , and day 8 was regarded as the second observation time point $t_{2}$ :
 
$R_{1}、A_{1}$ ← Calculate the mycelium radius and mycelium area at $t_{1}$ ;
 
$R_{2}、A_{2}$ ← Calculate the mycelium radius and mycelium area at $t_{2}$ .
**Output:** Use Eqs 1–5 to obtain five indexes of the mycelium outline ( R , L , A , $V_{R}$ , and $V_{W}$ ).

**FIGURE 5:**
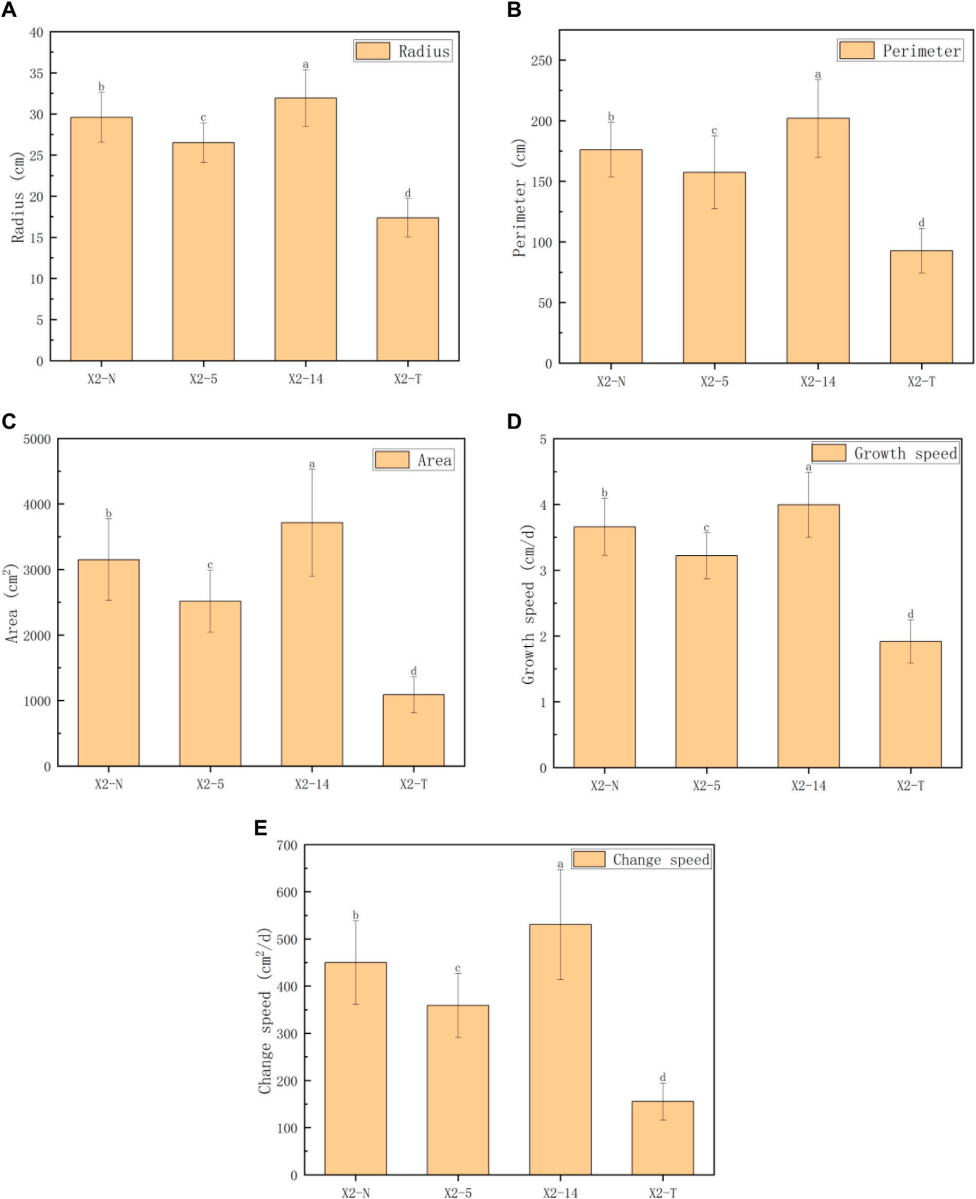
Comparison of five phenotypic characteristic indexes on the mycelial outline. **(A)** Radius (cm), **(B)** perimeter (cm), **(C)** area (cm^2^), **(D)** growth speed (cm/d), and **(E)** change speed (cm^2^/d). 1) The horizontal axis represented the four groups of mycelia, and the vertical axis represented each phenotypic data index; 2) all characteristic data were recorded on day 8 after subcultures and subject to the mean ±SD ( n=100 ) in each group; and 3) values with superscripts $\left\{a,b,c,d\right\}$ denoted significant differences across index rows ( p=0.05 ).

The five indices of the mycelial outline, namely, the mycelial radius, perimeter, area, growth rate, and change speed, were calculated to analyze the morphological and phenotypic characteristics (Hu et al., 2022), and the obtained results are shown in Table 1; Figure 5.

**TABLE 1:** Phenotypic characteristic indices of the mycelial outline.

Index	X2-N	X2-5	X2-14	X2-T	F	*p*
Radius (cm)	29.59 ± 3.04^b^	26.50 ± 2.44^c^	31.93 ± 3.46^a^	17.38 ± 2.32^d^	100.30	<0.001
Perimeter (cm)	176.10 ± 22.54^b^	157.39 ± 30.09^c^	201.94 ± 31.99^a^	92.68 ± 18.42^d^	62.66	<0.001
Area (cm^2^)	3,150.30 ± 621.31^b^	2,515.08 ± 474.53^c^	3,715.42 ± 816.76^a^	30.32 ± 33.55^d^	75.62	<0.001
Growth speed (cm/d)	3.66 ± 0.43^b^	3.22 ± 0.35^c^	4.00 ± 0.49^a^	1.92 ± 0.33^d^	100.30	<0.001
Change speed (cm^2^/d)	449.93 ± 88.76^b^	359.19 ± 67.79^c^	530.66 ± 116.68^a^	155.32 ± 39.25^d^	75.63	<0.001

**Note: 1)** All characteristic data were recorded on day 8 after subcultures and subject to the mean ±SD ( n=100 ) in each group; 2) values with superscripts $\left\{a,b,c,d\right\}$ denoted significant differences across index rows ( p=0.05 ); 3) all the comparison data were checked using the Bonferroni correction.

##### 2.2.1.1 Radius

$$R=\frac{Z_{L}+Z_{S}}{n_{p}}\times \pi R_{p},$$ (1)

where R is the mycelial radius; $Z_{L}$ and $Z_{S}$ are the number of pixels on the horizontal and vertical axes of the mycelium, respectively; and $n_{p}$ and $R_{p}$ are the number of pixels on the border of the Petri dish and the measured diameter of the petri dish, respectively.

##### 2.2.1.2 Perimeter

$$L=\frac{n_{v}}{n_{p}}\times 2\pi R_{p},$$ (2)

where L is the perimeter of the mycelium, $n_{v}$ is the number of pixels on the border of the mycelium, and $n_{p}$ is the number of pixels on the border of the Petri dish.

##### 2.2.1.3 Area

$$A=\frac{N_{V}}{N_{P}}\times \pi R_{p}^{2},$$ (3)

where A is the mycelial area, $N_{V}$ is the number of pixels of the Petri dish, and $N_{P}$ is the number of pixels of the mycelium.

##### 2.2.1.4 Growth rate

$$V_{R}=\frac{R_{2}-R_{1}}{t_{2}-t_{1}},$$ (4)

where $V_{R}$ is the growth rate of the mycelium, $R_{1}$ is the mycelial radius of $t_{1}$ observation time points, and $R_{2}$ is the mycelial radius of $t_{2}$ observation time points.

##### 2.2.1.5 Change speed

$$V_{W}=\frac{A_{2}-A_{1}}{t_{2}-t_{1}},$$ (5)

where $V_{W}$ is the change in mycelial growth and $A_{1}$ and $A_{2}$ are the mycelium areas at two observation time points.

#### 2.2.2 Texture processing methods

The steps of processing the phenotypic characteristics of the mycelium concerning texture morphology are summarized as follows:

Algorithm 2. The texture processing methods.

**Input:** The gray value of the original image of the mycelium is 0–255
1) $W_{F}$ ← Use the ratio of mycelial area $N_{V}$ and Petri dish area $N_{P}$ to calculate the mycelial coverage, as shown in Eq. 6;
2) $W_{P}$ ← Use the square of the ratio of mycelial perimeter L and the mycelial area A to calculate the mycelial roundness, as shown in Eq. 7;
3) $W_{C}$ ← Use the contrast feature quantity of the gray-scale co-occurrence matrix to calculate the mycelial groove depth, as shown in Eq. 8;
4) $W_{B}$ ← Use the gray value at the axis $\left(x_{g1},y_{g1}\right)$ to calculate mycelial density, as shown in Eq. 9;
5) $W_{S}$ ← Use the density at time $t_{2}$ and $t_{1}$ to calculate the density change, as shown in Eq. 10;
**Output:** Five indices of mycelial texture morphology, $W_{F}$ , $W_{P}$ , $W_{C}$ , $W_{B}$ , and $W_{S}$ .

**FIGURE 6:**
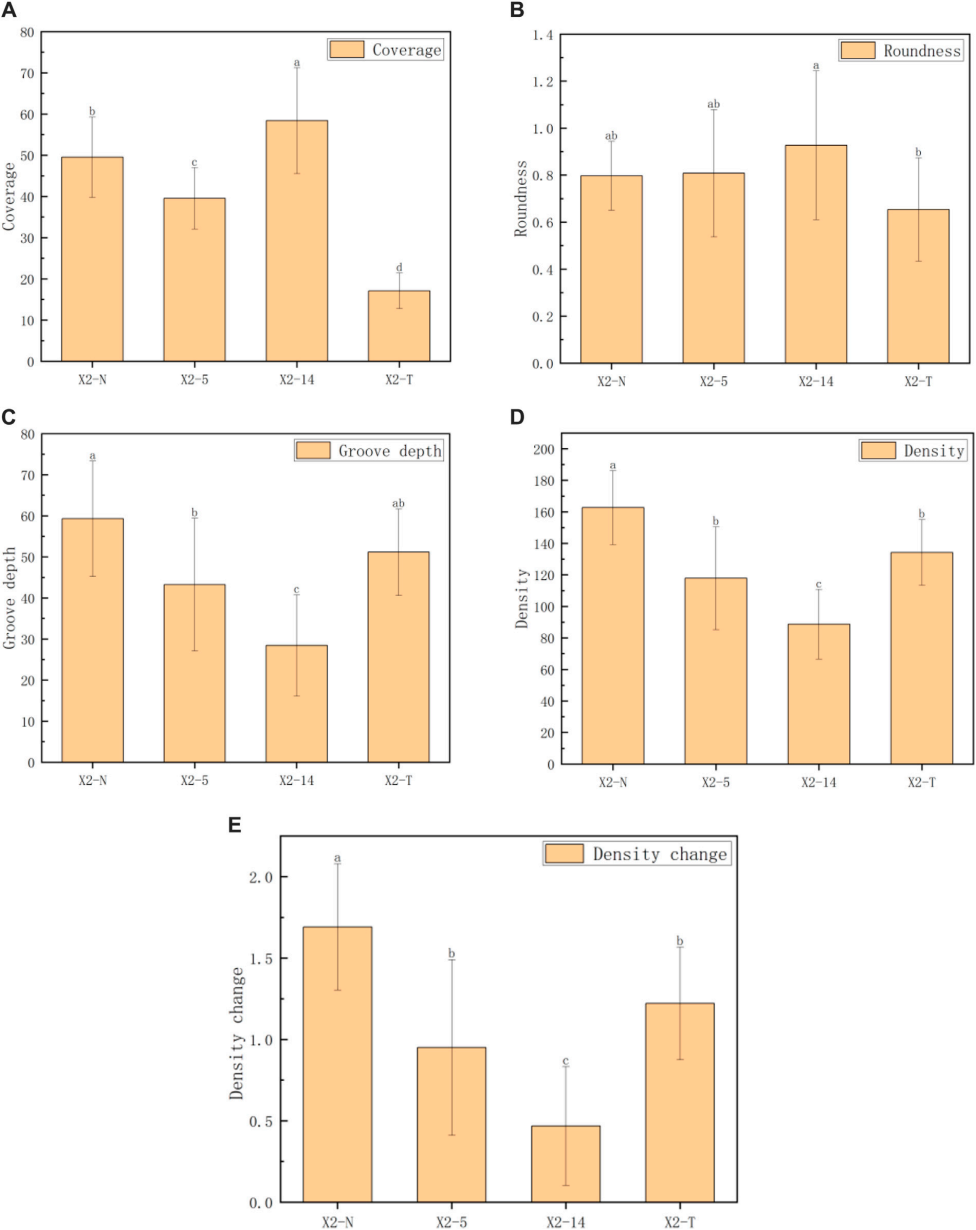
Comparison of five indexes of the mycelial texture. **(A)** Coverage, **(B)** roundness, **(C)** groove depth, **(D)** density, and **(E)** density change. 1) The horizontal axis represented the four groups of mycelia, and the vertical axis represented each phenotypic data index; 2) all characteristic data were recorded on day 8 after subcultures and subject to mean ±SD ( n=100 ) in each group; 3) values with superscripts $\left\{a,b,c,d\right\}$ denoted significant differences across index rows ( p=0.05 ).

The five indices of the mycelial texture, namely, coverage, roundness, groove depth, density, and density change, were computed to analyze the morphological and phenotypic characteristics (Hu et al., 2022), and the obtained results are shown in Table 2; Figure 6.

**TABLE 2:** Phenotypic characteristic indices of the mycelial texture.

Index	X2-N	X2-5	X2-14	X2-T	F	*p*
Coverage	49.52 ± 9.77^b^	39.53 ± 7.46^c^	58.40 ± 12.84^a^	17.10 ± 4.32^d^	75.62	<0.001
Roundness	0.80 ± 0.15^ab^	0.81 ± 0.27^ab^	0.93 ± 0.32^a^	0.65 ± 0.22^b^	4.12	0.009
Groove depth	59.30 ± 14.05^a^	43.24 ± 16.16^b^	28.43 ± 12.35^c^	51.15 ± 10.54^ab^	19.16	<0.001
Density	162.65 ± 23.46^a^	117.89 ± 32.62^b^	88.71 ± 22.07^c^	134.24 ± 20.89^b^	30.13	<0.001
Density change	1.69 ± 0.39^a^	0.95 ± 0.54^b^	0.47 ± 0.37^c^	1.22 ± 0.35^b^	30.13	<0.001

**Note: 1**. All characteristic data were recorded on day 8 after subcultures and subject to the mean ±SD ( n=100 ) in each group; 2) values with superscripts $\left\{a,b,c,d\right\}$ denoted significant differences across index rows ( p=0.05 ); 3) all the comparison data were checked by using the Bonferroni correction.

##### 2.2.2.1 Coverage

$$W_{F}=\frac{N_{V}}{N_{P}}\times \pi R_{p}^{2},$$ (6)

where $W_{F}$ is the coverage of the mycelium, $N_{V}$ is the number of pixels of the Petri dish, $N_{P}$ is the number of pixels of the mycelium, and $R_{p}$ is the measured diameter of the Petri dish.

##### 2.2.2.2 Roundness

$$W_{P}=\frac{L^{2}}{4\pi A},$$ (7)

where $W_{P}$ is the roundness of the mycelium, L is the perimeter of the mycelium, and A is the mycelial area.

##### 2.2.2.3 Groove depth

$$W_{C}=\sum_{g_{1}=1}^{N_{1}}\sum_{g_{2}=1}^{N_{2}}{\left(g_{1},g_{2}\right)}^{2}P\left(g_{1},g_{2}\right),$$ (8)

where $N_{1}$ is the number of pixels between the minimum and maximum values along the horizontal axis, $N_{2}$ is the number of pixels between the minimum and maximum values along the vertical axis, $g_{1}$ and $g_{2}$ are the brightness values of the pixels, and P is the total number of occurrences.

##### 2.2.2.4 Density

$$W_{B}=\frac{g_{1}+g_{2}+...+g_{n}}{f\left(x_{g1},y_{g1}\right)+f\left(x_{g2},y_{g2}\right)+...+f\left(x_{gn},y_{gn}\right)},$$ (9)

where $W_{B}$ is the density of the mycelium and $f\left(x_{g1},y_{g1}\right)$ is the gray value at the axis $\left(x_{g1},y_{g1}\right)$ .

##### 2.2.2.5 Density change

$$W_{S}=\frac{W_{B2}-W_{B1}}{t_{2}-t_{1}},$$ (10)

where $W_{S}$ is the density change and $W_{B2}$ and $W_{B1}$ denote the density at time $t_{2}$ and $t_{1}$ , respectively.

### 2.3 Methods for extracting enzyme activity from the mycelium

#### 2.3.1 Extraction method for cellulase activity

The activated mycelium was placed in a centrifuge tube using a hole puncher with a diameter of 5 mm and was then stored in a refrigerator at 4 °C . Approximately 0.1 g of mycelial tissue was measured, to which 1 mL of extract was added. The mixture was homogenized in an ice bath and then centrifuged at 8,000 g for 10 min at 4 °C . The supernatant was carefully extracted and stored on ice for subsequent use (Marei et al., 2012).

We adapted the anthrone colorimetric method to quantify the reducing sugar content generated through the degradation of sodium carboxymethyl cellulose, which was catalyzed by CL (Wood and Bhat, 1988). The procedure involved shaking the sample at 37 °C for 1 h and then placing it in a 90- °C water bath for 15 min. Subsequently, we centrifuged the sample at 8,000 g at 25 °C for 10 min after cooling, collected the supernatant, and mixed it to prepare the saccharification solution. This solution was then incubated in a 90 °C water bath for 10 min. After cooling, we took 200 μL of the reaction solution and measured the absorbance value A at 620 nm using a 96-well plate and then calculated $∆A=A^{+}\;-A^{-}$ , where $A^{+}$ and $A^{-}$ denote the measurement and control tubes, respectively. It was noted that these reducing sugars can react with the anthrone reagent to form a blue–green complex, the intensity of which is proportional to the concentration of the reducing sugar. By measuring the color intensity after the reaction, the activity of cellulase can be indirectly calculated, that is, the amount of reducing sugar produced by cellulose breakdown per unit time.

**FIGURE 7:**
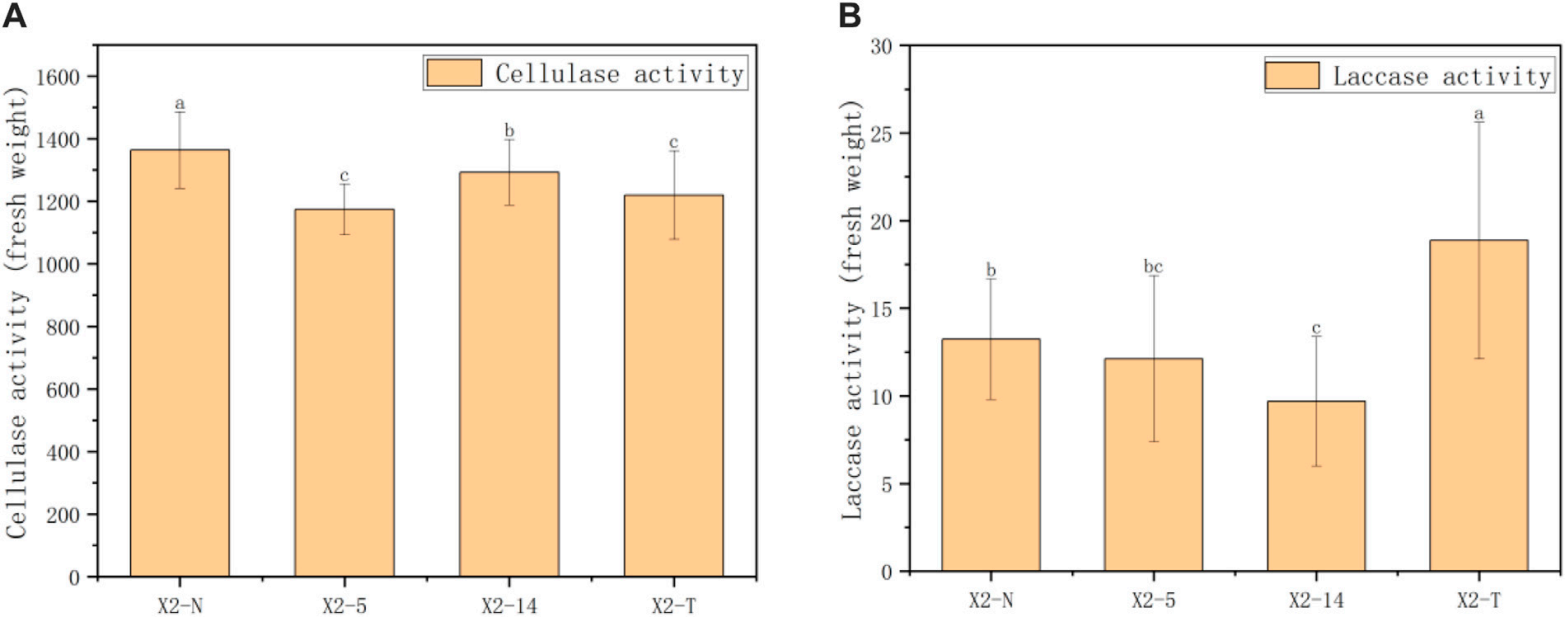
Results of enzyme activity in four groups of mycelia. **(A)** Results of cellulase activity in four groups of mycelia and **(B)** results of laccase activity in four groups of mycelia. 1) The horizontal axis represented the four groups of mycelia, and the vertical axis represented the enzyme activity in units of fresh weight; 2) all characteristic data were recorded on day 8 after subcultures and subject to the mean ±SD ( n=100 ) in each group; 3) values with superscripts $\left\{a,b,c,d\right\}$ denoted significant differences across index rows ( p=0.05 ).

The regression equation for determining standard conditions was given by y=2.5090x−0.0462 , where x is the standard concentration ( mg/mL ) and y is the absorbance value. The catalytic production of 1 μg of glucose per g of tissue per minute is defined as a unit of enzyme activity. Then, based on the fresh weight of the sample, we calculated cellulase activity $V_{CL}\left(\mu g/\min/g\right)$ as shown below, and the results are given in Figure 7A.

$$V_{CL}=\left[1000\times \left(∆A+0.0462\alpha \right)÷2.509\times \beta_{f}\right]÷\left(W\times \beta_{s}÷\beta_{t}\right)÷T=79.6\times \left(∆A+0.0462\beta_{f}\right)÷W,$$ (11)

where $\beta_{f}$ denotes the total volume of the reaction, $\beta_{s}$ is the reaction volume of samples, $\beta_{t}$ is the extra volume of the extraction, T is the reaction time, and W is the sample mass. In this test, we gave the following parameters: $\beta_{f}=0.6\;mL$ , $\beta_{s}=0.05\;mL$ , $\beta_{t}=1\;mL$ , and T=60 min .

#### 2.3.2 Extraction method for laccase activity

The activated mycelium was placed in a centrifuge tube using a hole puncher with a diameter of 5 mm and was then stored in a refrigerator at 4 °C . Approximately 0.1 g of mycelial tissue was measured, to which 1 mL of the extract was added. The mixture was homogenized in an ice bath and then centrifuged at 13,000 g for 30 min at 4 °C . The supernatant was carefully extracted and stored on ice for subsequent use.

Laccase decomposes the substrate ABTS to produce ABTS free radicals, which have a significantly higher absorbance coefficient at 420 nm than the substrate ABTS. By determining the increase rate of ABTS free radicals, the activity of laccase can be calculated. The reaction mixture was cooled to room temperature after 20 min in a water bath at 60 °C . We took 200 μL of the reaction solution and measured the absorbance value A at 420 nm using a 96-well plate and then calculated $∆A=A^{+}\;-A^{-}$ , where $A^{+}$ and $A^{-}$ denote the measurement and control tubes, respectively. The amount of enzyme required to oxidize 1 nmol substrate ABTS per gram of sample per minute was defined as one unit of enzyme activity. Then, based on the fresh weight of the sample, we calculated cellulase activity $V_{Laccase}\left(nmol/\min/g\right)$ as shown below, and the results by using Eq. 12 are given in Figure 7B.

$$V_{Laccase}=\left[\frac{∆A}{\varepsilon \times d}\times \beta_{f}÷\left(\beta_{s}\times W÷\beta_{t}\right)\right]÷\left(W\times \beta_{s}÷\beta_{t}\right)÷T=78.52\times ∆A÷W,$$ (12)

where ε denotes the millimolar extinction coefficient of ABTS, d is the light diameter of the cuvette, $\beta_{f}$ denotes the total volume of the reaction, $\beta_{s}$ is the reaction volume of samples, $\beta_{t}$ is the extra volume of the extraction, T is the reaction time, and W is the sample mass. In this test, we gave the following parameters: ε=36 L/mmol/cm , d=0.5 cm , $\beta_{f}=0.3\;mL$ , $\beta_{s}=0.045\;mL$ , $\beta_{t}=1\;mL$ , and T=20 min .

## 3 Results

### 3.1 Results of the mycelial outline

For convenience, four groups of mycelium, namely, the non-degraded and degraded mycelium and the 5th and 14th subcultures, were denoted as X2-N, X2-T, X2-5, and X2-14, respectively. We took 100 samples selected from each group. A total of 400 samples were photographed and recorded on day 8 after inoculation. We precisely computed the outline morphology of the mycelium by using Algorithm 1, resulting in a total of five phenotypic characteristic indices: radius, perimeter, area, growth speed, and change speed. We adopted the one-way ANOVA method, and the mean and standard deviation (SD) of the five indices for the four groups of mycelium are shown in Table 1. The results showed that there were significant statistical differences in the five indices among the four groups (all P<0.001 ). The pairwise comparison of each index showed that all five indices of X2-T related to the outline morphology are lower than those of the X2-N and X2-5 and X2-14.

Figure 5 shows the performance trends of the five indices on the phenotypic characteristics of the mycelial outline (radius, perimeter, area, growth rate, and change speed), which were basically consistent. The growth rate of the mycelium weakened from the activation stage to X2-5, but it accelerated from X2-5 to X2-14. However, for X2-T, there was a significant downward trend in all aspects.

### 3.2 Results of the mycelial texture

For the 400 existing samples, we accurately calculated the texture morphology of the mycelium by using Algorithm 2, resulting in five indices of phenotypic characteristics: coverage, roundness, groove depth, density, and density change. We took the one-way ANOVA method, and the mean and SD of the five indices of the four groups of mycelium are given in Table 2. The results showed that there were statistical differences in the five indices among the four groups (all P<0.05 ). Among them, the difference in the mycelial roundness data was the smallest ( P=0.009 ), and the differences among the other indices were more significant ( P<0.001 ). The pairwise comparison of each index showed that X2-T had the lowest mycelium coverage, while X2-14 had the worst performance in terms of groove depth, density, and density change.

The coverage indices given in Figure 6 were generally consistent. The roundness of the four groups of mycelia showed little difference. However, as the subculture times increase, the groove depth, mycelial density, and their rate of changes significantly decreased, even falling below X2-T. This phenomenon suggested that during the process, a faster growth rate in the subculture leads to sparser mycelium texture, darker coloration, and shallower grooves. On the contrary, X2-T exhibited the lowest coverage due to its extremely slow growth rate but maintained relatively completed roundness, and the mycelial texture was still white and dense with obvious grooves.

### 3.3 Results of enzyme activity

In this study, 30 samples were selected from X2-N, X2-5, X2-14, and X2-T. The anthrone colorimetric method was used to calculate the index of cellulase activity in the unit of mycelium mass using Formula 11. The comprehensive statistical results of these indexes through one-way ANOVA showed that there were statistical differences in cellulase activity among the four groups ( P<0.001 ). As shown in Figure 7A, during the process of subculture, the cellulase activity was subject to the characteristics of decreasing and then increasing, and the cellulase activity of the degraded mycelium was lower than that of X2-N. Furthermore, as shown in Figure 7B, the laccase activity was most active in the degraded mycelium.

Comparing Figures 5–7 shows that the cellulase activity trend of the mycelium was basically consistent with the analysis results of five indices related to its mycelium outline, as well as the analysis results of the two indices of mycelium coverage and roundness in its texture morphology. However, the laccase activity of the strain was different from the 10 indices in the outline and texture of the mycelium, and contrarily, the laccase activity of the degraded mycelium was particularly significant. Therefore, from the physiological perspective of the mycelium, the cellulase activity of this mycelium can be consistent with the phenotypic characteristic indices of the mycelium. Cellulase activity can be well used to verify the accuracy of digital image processing technology in the analysis of phenotypic characteristics.

## 4 Discussion

As a species of edible fungi with high yield and high economic value, the analysis of the phenotypic characteristics of *P. geesteranus* currently involves a lot of manual repeated operations, mainly including the average growth rate, color, texture, density, and edge uniformity, measured and calculated by a manual ruler or experience (Cardini et al., 2020). By applying digital image technology to the analysis of the mycelium, its phenotypic data can not only improve the accuracy of phenotypic characteristics obtained by manual measurement but also convert qualitative phenotypic characteristics into objective ones that can be qualitatively and quantitatively analyzed (Hardy et al., 2017). It has been shown that the use of digital image processing technology is regarded as an automated mean to extract and analyze the phenotypic characteristics of the mycelium, which has obvious advantages (Phoulady et al., 2016).

In order to better analyze the phenotypic characteristics of *P. geesteranus*, digital image processing technology has made certain progress in the following aspects (Alkhudaydi et al., 2019):

1) Image acquisition and preprocessing: High-quality image acquisition equipment and preprocessing techniques are used to obtain the enhanced images of the growth process of *P. geesteranus.*
2) Feature extraction: Relevant features related to the phenotype of *P. geesteranus* are extracted from the preprocessed images. These features can include shape, color, and texture (Yang et al., 2024).
3) Algorithm design and analysis: Utilizing algorithms and mathematical formulas to digitally calculate the phenotypic characteristics of *P. geesteranus* (Zhai et al., 2019).
4) Application and expansion: Combining technologies from other fields with digital image processing to achieve more efficient and accurate phenotypic analysis of *P. geesteranus* (Wei et al., 2021).

In our study, the statistical analysis results of the above phenotypic characteristics showed that during the subculture process of the mycelium, the growth rate of the mycelium showed a trend of slowing down at first and then accelerating, and the texture quality of the mycelium decreased with the acceleration of their growth rate. At the same time, the growth ability of the degraded mycelium was definitely the weakest. Therefore, in the early breeding stage of the mycelium, we could efficiently eliminate those degraded mycelia with slow production speed from the outline indices, and we also eliminated those mycelia with uneven texture and sparse density from the texture indices. Thus, among these two types of phenotypic characteristics, the mycelium with better growth, higher quality, and stronger vitality could be quickly identified for cultivating *P. geesteranus*.

In summary, there is still considerable room for development in the digital image processing research of *P. geesteranus* phenotypic analysis. In this paper, four groups of mycelia, X2-N, X2-5, X2-14, and X2-T, were formulated by using digital image processing technology from its outline and texture morphology. However, different edible fungus species had individual differences, and their phenotypic characteristics were also different in the macroscopic view. Therefore, in the field of research on the phenotypic characteristics of edible fungi, it is necessary to combine the characteristics of different mycelia to address proper algorithms and analysis schemes of phenotypic characteristics (Zhang et al., 2020).

## 5 Conclusion

This paper studied the phenotypic characteristics of the mycelium of *P. geesteranus* by using image recognition technology. We found that the mycelium was analyzed from the outline and texture shapes. In the outline shape, five indexes, namely, mycelium radius, perimeter, area, growth rate, and change speed, were calculated to obtain the results of quantitative analysis. In texture shape, five indices, namely, mycelial coverage, roundness, groove depth, density, and density change, were calculated to obtain the results of qualitative analysis. All indices of the degraded mycelium were significantly lower than those of the non-degraded mycelium, and the obtained analysis results showed large statistical differences ( P<0.05 ). Moreover, these analysis results were further verified by using cellulase activity. It could be observed that during the subculture process of the mycelium, the growth rate of the mycelium showed a trend of slowing down at first and then accelerating, and the texture quality of the mycelium decreased with the acceleration of its growth rate. At the same time, the growth ability of the degraded mycelium was definitely the weakest. Our results revealed a close relationship between phenotypic characteristics and mycelial quality, providing a rapid and accurate method for strain selection in the early breeding stage of *P. geesteranus*.

## Data Availability

The original contributions presented in the study are included in the article/Supplementary Material; further inquiries can be directed to the corresponding author.
